# Interval From Simulation Imaging to Treatment Delivery in SABR of Lung Lesions: How Long is Too Long for the Lung?

**DOI:** 10.1016/j.adro.2022.101132

**Published:** 2022-11-30

**Authors:** Gilles Colin, Selma Ben Mustapha, Nicolas Jansen, Philippe Coucke, Laurence Seidel, Patrick Berkovic, Levente Janvary

**Affiliations:** a Department of Radiation Oncology, University Hospital of Liège, Liège, Belgium; b Department of Biostatistics, University Hospital of Liège, Liège, Belgium; c Department of Radiation Oncology, University Hospitals Leuven, Leuven, Belgium; d Department of Radiation Oncology, National Institute of Oncology, Budapest, Hungary

## Abstract

**Purpose:**

The purpose of this study was to evaluate the effect of delay between planning computed tomography (CT) used as a basis for treatment planning and the start of treatment (delay planning treatment [DPT]), on local control (LC) for lung lesions treated by SABR.

**Methods and Materials:**

We pooled 2 databases from 2 monocentric retrospective analysis previously published and added planning CT and positron emission tomography (PET)–CT dates. We analyzed LC outcomes based on DPT and reviewed all available cofounding factors among demographic data and treatment parameters.

**Results:**

A total of 210 patients with 257 lung lesions treated with SABR were evaluated. The median DPT was 14 days. Initial analysis revealed a discrepancy in LC as a function of DPT and a cutoff delay of 24 days (21 days for PET-CT almost systematically done 3 days after planning CT) was determined according to the Youden method. Cox model was applied to several predictors of local recurrence–free survival (LRFS). Univariate analysis showed LRFS decreasing significantly related to DPT ≥24 days (*P* = .0063), gross tumor volume, and clinical target volume (*P* = .0001 and *P* = .0022), but also with the presence of >1 lesion treated with the same planning CT (*P* = .024). LRFS increased significantly with higher biological effective dose (*P* < .0001). On multivariate analysis, LRFS remained significantly lower for lesions with DPT ≥24 days (hazard ratio, 2.113; 95% confidence interval, 1.097-4.795; *P* = .027).

**Conclusions:**

DPT to SABR treatment delivery for lung lesions appears to reduce local control. Timing from imaging acquisition to treatment delivery should be systematically reported and tested in future studies. Our experience suggests that the time from planning imaging to treatment should be <21 days.

## Introduction

Time plays a crucial role in radiation oncology (RO), sometimes in unexpected ways (FLASH–radiation therapy [RT],^1^ chrono-RT^2^). In the treatment of cancer, by definition a progressive disease, avoidance of delays is essential.^3^^,^^4^ This was recently challenged during the COVID-19 pandemic.^5^

In SABR, it is obvious that the delay between planning computed tomography (CT) used as a basis for treatment planning and the start of treatment (delay planning treatment [DPT]) must be as short as possible. This reduces changes in the lesion (size and shape) to be treated and/or the patient's anatomy, thus increasing the precision of the delivered treatment. DPT is also a required period for target volume and organs at risk identification, treatment plan preparation and pretreatment quality control.^6^

Causes of long DPT are numerous and not fully discussed in this article such as the complexity of treatment plans, increased demand of SABR,^7^ treatment machine breakdown and patient's intercurrent pathologies. At the level of the treatment team both oversight or staffing problems can play a role.

Ongoing trials are investigating the efficacy of SABR for oligometastatic disease in up to 10 lesions.^8^ If it is not possible to treat all lesions in the same treatment session, the choice of the best sequence (simultaneous, alternating, sequential) is still partly unknown.^9^ In case of sequential treatment, the DPT for the last treated lesion could become too long.

But “how long is too long”? For brain metastases RT, a retrospective analysis addressed this question and suggested a maximal delay of 14 days between the MRI scan and the start of stereotactic radiation surgery (SRS).^10^ Moreover, a prospective analysis of 69 lesions (including 15 resection cavities) found that in 46% of cases, an interval of <7 days between the planning MRI and a second MRI performed 24 hours before the treatment required a replanning. This percentage increased to 62% of cases with an interval between 8 and 14 days.^11^ Although it is reasonable to suppose that replanning does not always mean better local control (LC), this increase in rates remains questioning*.*

To the best of our knowledge, there is no specific data on DPT in SABR for pulmonary lesions. Moreover, the study protocols like the Radiation Therapy Oncology Group (RTOG) 0915 study for primary lung lesions^12^ and the SABR-COMET studies for secondary lesions,^8^^,^^13^^,^^14^ do not report these delays. A recent American Society for Radiation Oncology white paper on the safety of SRS/SABR reiterates the need to specify these temporal criteria in trials and recommends not to exceed a DPT of 14 days for SRS.^15^ To assess the effect of DPT on LC for lung lesions, we retrospectively analyzed patients treated by SABR with the CyberKnife (CK) system. Our hypothesis was that a long DPT will have a negative effect on LC independently of other variables such as volume or prescription dose.

## Methods and Materials

### Patient selection

In 2020, Berkovic et al published the results of a monocentric retrospective analysis of 104 patients and 132 metastatic lung lesions treated with SABR on CK in the setting of oligorecurrent disease between May 2010 and March 2016.^16^ In 2017, Janvary et al published a retrospective analysis of 130 patients and 160 lung lesions (primary, recurrent, or metastatic) treated consecutively with SABR at the same center and on the same treatment machine between April 2010 and June 2012.^17^

We pooled these 2 databases, removed duplicates by keeping the lesion with the longest follow-up, and added planning CT and positron emission tomography (PET)–CT dates. For the few identified conflicting data, a review of the institutional records of the patients was performed.

Two patients with lung metastases arising from adenoid cystic carcinoma of the salivary glands were removed because the slow progression of this disease could limit the effect of a large DPT on LC and complicate follow-up. Ultimately, 210 patients and 257 lung lesions were analyzed.

### Statistical analyses

DPT duration was calculated from existing database items. Results were expressed as means, standard deviations or medians (Q1-Q3) for quantitative variables and as numbers and percentages for categorical variables. To determine the best cutoff value for DPT based on LC, we used the Youden method. Means between the 2 groups thus defined were compared with a Student *t* test and proportions with a χ^2^ test. To normalize their distribution, some variables were log-transformed. Local recurrence–free survival (LRFS) was examined using Cox regression models. Multivariate model with stepwise selection was also applied. The hazard ratio (HR) and its 95% confidence interval were reported. LRFS was plotted using Kaplan-Meier curves. Results were considered significant at the 5% significance level (*P* < .05). All statistical analyses were carried out by SAS version 9.4 (SAS Institute, Cary, NC) and figures by R version 4.1.1.

## Results

### Demographic data and treatment parameters

A total of 210 patients and 257 treated lesions were included in the analysis. Key demographic and treatment data are available in the source articles. Initial analysis revealed a discrepancy in LC as a function of DPT. According to the Youden method, a cutoff of 24 days was set. This cutoff shows very good specificity (88.1%) but low sensitivity (25.0%). Demographic and treatment parameters are reported in Table 1 in each arm: DPT <24 days (arm A) and DPT ≥24 days (arm B).

**Table 1:** Demographic and treatement parameters

		Arm A	Arm B	*P* value
Parameters		DPT <24 d	DPT ≥24 d	
**Patients and lesions**				
Number of lesions		219	38	
Age (y), mean ± SD		67.8 ± 11.3	67.2 ± 12	.78
GTV (cm³), mean ± SD		9.72 ± 14.9	9.2 ± 11.4	.81
Lesion				.63
	Primary	72 (33%)	11 (29%)	
	Secondary	147 (67%)	27 (71%)	
Origin of primary tumor				.76
	Gastrointestinal	71 (48%)	12 (44%)	
	Lung	44 (30%)	10 (37%)	
	Other	32 (22%)	5 (19%)	
Chemotherapy for primary		116 (79%)	24 (89%)	.35
**Secondary lesions**				
Number of secondary lesions		147	27	
Chemotherapy for previously treated secondary lesions		54 (37%)	11 (41%)	.69
Radiation therapy for previously treated secondary lesions		38 (26%)	9 (33%)	.42
≥2 lines of chemotherapy before current treatment		22 (15%)	6 (22%)	.35
≥2 lesions treated with the same planning CT		34 (23%)	14 (52%)	.0021
**Treatment**				
BED (Gy), mean ± SD		150 ± 39.4	146 ± 39.8	.54
PTV (cm³), mean ± SD		29.8 ± 29.7	30.1 ± 23.3	.95
Number of fractions				.80
	3	160 (73.1%)	27 (71.1%)	
	5	59 (26.9%)	11 (28.9%)	
Presence of real-time tumor tracking		114 (52%)	13 (34%)	.042

Abbreviations: BED = biological equivalent dose; CT = computed tomography; GTV = gross tumor volume; PTV = planning target volume; SD = standard deviation.

BED with α/β = 10 Gy.

A total of 219 (85.2%) lesions were treated in arm A and 38 in arm B. There was no difference between the 2 groups in age, gross tumor volume (GTV) and planning target volume (PTV), biological effective dose (BED), number of fractions, or percentage of treatment of primary or secondary lesions. However, tracking technique (spine tracking versus real-time tumor tracking) was used more frequently in the short delay group (*P* = .042). Regarding SABR of metastatic lesions, there was no difference in the percentage of pulmonary, digestive, or other primary origin. There was no difference in the percentage of use of previous chemotherapy or radiation therapy. Treatment of multiple lesions with the same planning CT (≥2) is significantly more frequent in arm B (≥24 days; *P* = .0021). In both arms, the treated volumes were determined using the same margins: 3 mm from GTV to clinical target volume (CTV).

### Delay planning treatment

**Figure 1:**
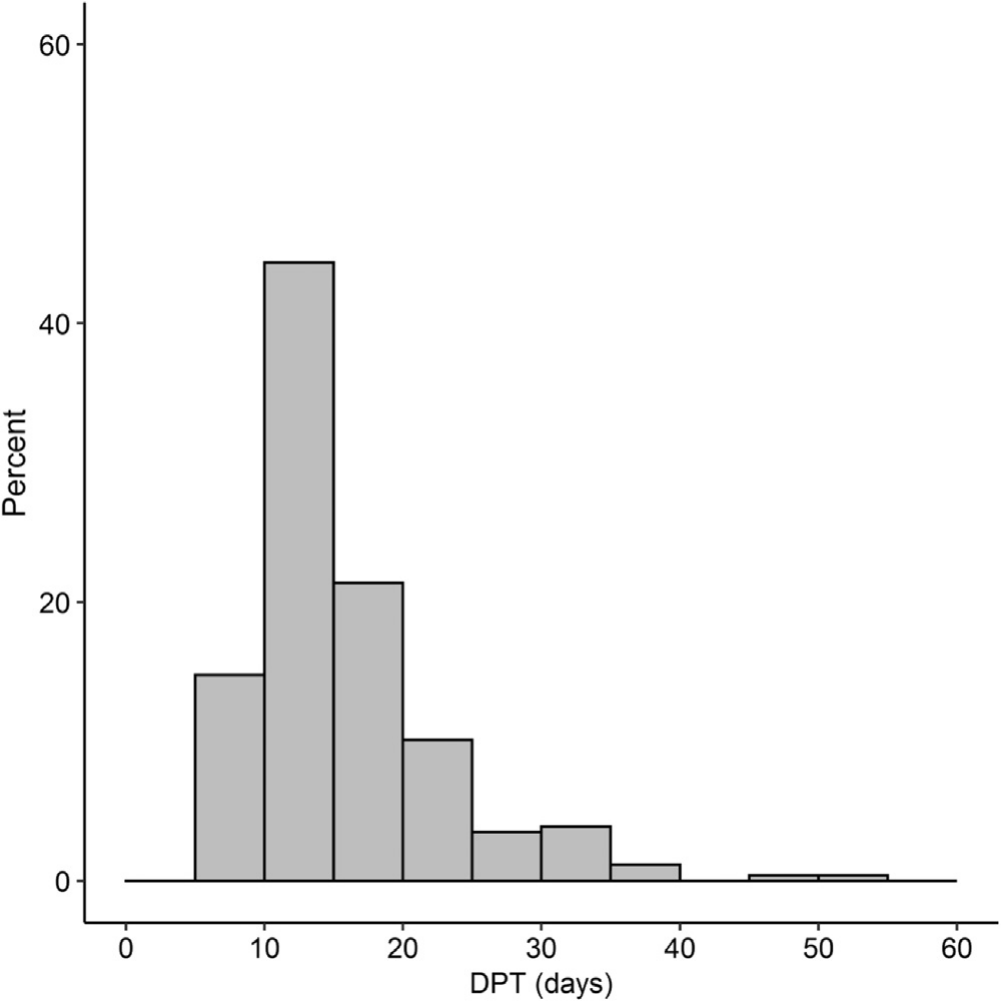
Frequency histogram of delay planning treatment (DPT) expressed in days.

The median time from planning CT to first day of treatment was 14 days (Q1-Q3, 11-19 days). Figure 1 shows the frequency histogram of DPT expressed in days. Almost all patients had planning PET-CT 3 days after planning CT and the histogram is simply shifted by 3 days. This excludes the delay “PET to treatment" as a confounding factor and this delay is therefore not considered further.

### LC and LRFS by DPT

The median DPT was 13 days (11-18 days) for locally controlled lesions versus 14 days (12-23 days) for LR lesions. Using the Cox model, the risk of LR increased significantly with DPT, with a HR of 2.11 (*P* = .029). Cox model was applied to several predictors of LRFS (Table 2). Univariate regression analysis was performed for different variables of interest, such as time course (DPT and cutoff delay), BED, GTV, PTV, and presence of tumor real-time tracking or prior cytotoxic treatment (chemotherapy or radiation therapy).

**Table 2:** Univariate and multivariate analysis in Cox model for different explanatory variables of local recurrence

Parameters	HR	95% CI		*P* value
**Univariate Cox regression analysis**				
DPT (d, log)	2.113	1.081	4.129	.029
Arm B (≥24 d) versus arm A (<24 d)	2.328	1.269	4.271	.0063
BED (with α/β = 10 Gy)	0.986	0.980	0.992	<.0001
GTV (cm³, log)	1.490	1.214	1.828	.0001
PTV (cm³, log)	1.608	1.187	2.178	.0022
≥2 lesions treated with the same planning CT	2.044	1.098	3.806	.024
≥2 lines of chemotherapy before current treatment	1.738	0.873	3.460	.12
Presence of real-time tumor tracking: yes versus no	1.061	0.628	1.793	.82
Primary versus secondary lesions	0.732	0.399	1.341	.31
Radiation therapy for previously treated secondary lesions	1.628	0.865	3.064	.13
Chemotherapy for previously treated secondary lesions	1.005	0.542	1.862	.99
**Multivariate Cox regression analysis**				
Arm B (≥24 d) versus arm A (<24 d)	2.293	1.097	4.795	.027
BED (Gy)	0.987	0.979	0.995	.001
GTV (cm³, log)	1.363	1.065	1.746	.014
≥2 lesions treated with the same planning CT	2.127	1.061	4.263	.033

*Abbreviations*: 95% CI = 95% confidence interval of hazard ratio; BED = biological equivalent dose; CT = computed tomography; DPT = delay planning treatment; GTV = gross tumor volume; PTV = planning target volume.

BED with α/β = 10 Gy.

LR was found to be significantly related to time delay (DPT[log]: HR, 2.11; *P* = .029; DPT ≥24 days: HR, 2.33; *P* = .0063) and volume (GTV[log]: HR, 1.49; *P* = .0001; PTV[log]: HR, 1.61; *P* = .0022), but also increased with the presence of >1 lesion treated with the same planning CT (HR, 2.04; *P* = .024). LR decreased with a higher BED (HR, 0.99; *P* < .0001).

The multivariate Cox regression analysis showed that the following parameters were significant: the cutoff time of 24 days (HR, 2.29; *P* = .027), GTV(log) (HR, 1.34; *P* = .014), BED (HR, 0.99; *P* = .001), and the presence of >1 lesion to be treated with a single planning CT (HR, 2.13; *P* = .033).

### Survival curves for the local recurrence event

**Figure 2:**
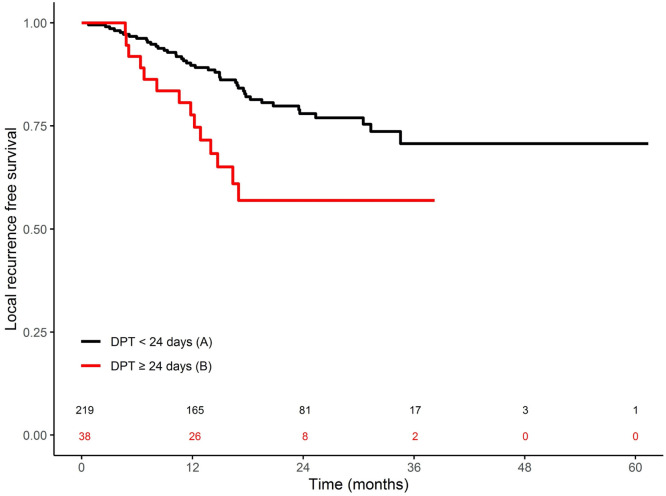
Kaplan-Meier curves for local recurrence–free survival with significantly greater local control for lesions with delay planning treatment (DPT) <24 days (*P* = .0063).

Figure 2 shows the Kaplan-Meier curves of LRFS in each arm (A: DPT <24 days and B: DPT ≥24 days). There was a LC dropout in arm B (HR, 2.33; *P* = .0063). LRFS at 12 months was 89.7% for arm A and 77.7% for arm B.

## Discussion

DPT is an important period in RO. Obviously this delay should be as short as possible without compromising the quality of the process leading up to the treatment (a principle that could be called ASASA [as soon as safely achievable]). To reduce this period, online adaptive radiation therapy is a promising concept but technical and clinical challenges remain.^18^

To date, little is known about the safe maximal time interval between the planning CT and the start of treatment in SABR of primary and secondary lung lesions. Some guidelines recommend a maximum DPT of 14 days. This cutoff is based on retrospective data from the treatment of brain metastases with SRS.^15^ There are no clear recommendations for lung lesions, even in study protocols addressing this technique.^8^^,^^12^, ^13^, ^14^

Our retrospective analysis confirms the effect of DPT on LC for primary and secondary lung lesions. This effect withstands multivariate analysis including BED and tumor/target volume known to affect LC. Other available variables were also tested but do not significantly affect LC such as a tracking method or previous chemotherapy/radiation therapy treatment. The presence of tracking allows to assess whether the decrease of LC could be due to anatomic changes of the patient that make spinal tracking of CK less efficient. Previous cancer treatment could be a marker of radioresistance and/or rapid repopulation, which would explain lower LC.^19^

Determining a cutoff DPT with prospective testing of this delay is undesirable for obvious ethical considerations and has no clinical basis. The only way to investigate this DPT is therefore with retrospective data. In our analysis, a cutoff of 24 days was defined based on the Youden method. This cutoff shows a good specificity but poor sensitivity, which is not surprising because a short delay does not provide certainty of LC. It allows us to define 2 arms and observe a significant decrease in LC for patients in arm B with DPT ≥24 days. This is of course a maximum time frame from which a new simulation should be considered. With the 3 days gap between the planning CT and PET-CT, we recommend a maximum of 21 days between imaging used for treatment planning and the start of treatment. The recommended 14 days seem to be a clinically relevant choice while remaining pragmatic.^13^

Several hypotheses can explain the decrease in LC with DPT observed in this study. The main one concerns a geometric miss. Lesions can change both in volume and shape with time explaining decrease in LC. This problem could be exacerbated by the current “2.5D” image guidance during RT by the CK system, which makes volumetric assessment of the lesion difficult. A full soft-tissue 3-dimensional imaging system and a medical procedure to inspect these images at each fraction would not solve all problems (because microscopic disease is not considered) but could already detect (large) macroscopic changes.^20^

The evolution of neoplastic lesions remains very heterogeneous. It is known that tumor volume/growth plays a role on probability of tumor control.^21^^,^^22^ This raises the question of appropriate DPT depending on the type of lesion, histology, or previous treatments. For example, in primary lung tumors, Murai et al retrospectively analyzed the progression of stage I non-small cell lung cancers treated with SABR between diagnostic CT and planning CT. They showed a 2-fold longer doubling time for adenocarcinomas compared with squamous cell carcinomas.^23^ Another cause of geometric miss could be an anatomic change around the lesion. Concerning this geometric miss, the systemic CTV margin of 3 mm used for all of our patients could have compensated for some modifications, thus underestimating the effect of the delay. In most of the other series assessing SABR for lung lesions, the principle of GTV = CTV is often used.^8^^,^^12^, ^13^, ^14^

### Limitations

Several limitations exist in this study. First, it is an aggregation of retrospective studies with sometimes limited follow-up period. This can lead to inexact results or lack of robustness. To evaluate this problem, a rapid update of our data from LC based on available institutional imaging and pathology follow-up protocols was made. Ten additional LR in arm A (DPT <24 days) versus 7 in arm B (DPT ≥24 days) were found (unpublished data). Although this information is basically crude, it seems relevant to us given the relatively short follow-up time of the 2 source studies (median follow-up time of <2 years, 66 lesions <1 year). We note that 10 of the 17 identified recurrences occurred in the first year after SABR. This update increases the significance of all tests performed in this study. For example, HR increases from 2.11 (*P* = .029) to 2.94 (*P* = .0003) with a median DPT of 13 days (11-18 days) and 14 days (12-24 days), depending on LC and LR, respectively. To test the robustness of the analysis, we performed an identical analysis with the extreme values of DPT (over 5 weeks) removed. In this scenario, the cutoff delay remains at 24 days and the statistical analysis at this cutoff value remains significant.

A second limitation of this study concerns numerous confounding factors. We have seen that planning PET-CT was almost systematically done 3 days after the simulation CT, so this was not directly considered a factor. Another potential confounding factor could be that the longer DPT is associated to a more complicated treatment plan, which can be associated with a lower BED. A correlation test between the 2 variables showed a negative correlation but remained nonsignificant (*P* = .11). Furthermore, multivariate analysis accounting for BED and cutoff delay remained significant for the latter.

Regarding the dose, the volume and type of the lesions, the article by Janvary et al showed a better LC for smaller tumor volume, higher BED and for primary tumors compared with metastases.^17^ Berkovic et al showed a better LC for tumor volume, BED and for metastases of digestive origin compared with the "other" groups^16^ despite conflicting data in the literature.^24^ In our study, these different factors were well distributed between both arms.

For metastatic lesions, previous systemic treatments with chemo, immuno- or targeted therapies may act as radiomodulator agents and affect LC. Only information about previous chemotherapy was available and well balanced between both arms.

Another possibility would be a greater radiation resistance of lesions with a larger DPT, either acquired during this period (very hypothetical) or related to the fact that more than one lesion is more often treated in the case of a large DPT. Irradiation of multiple lesions is certainly the most important confounding factor. It can be considered as a cause of delay due to it being more frequent in arm B (DPT ≥24 days; *P* = .0021). However, a DPT ≥24 days remains significant even after adjusting for irradiation of multiple lesions. Identification of other causes of delay was not the aim of this article. Some of these can also be considered confounders (eg, deterioration of patients with change/disruption in breathing pattern).

Finally, 2 more arguments illustrate the complexity of the situation and the importance of DPT. These aspects are not discussed in this article but support our conclusions. First, cancer treatment care delay is a well-known problem.^4^ Delay may have an effect on the distant progression of the disease, especially if RT requires the therapeutic window of systemic treatments. It may also necessitate restaging and a different therapeutic approach. Second, in addition to macroscopic geometric miss, tumor change during the delay may result in a lower and less-uniform dose outside the (true) GTV and underdosage of microscopic disease with the risk of local and distant recurrence.^25^^,^^26^

## Conclusions

SABR of lung lesions is now part of routine clinical practice in many radiation therapy centers. The maximum DPT to avoid compromising LC is not known and limited data are available. Our experience reflects the period of the introduction of SABR in our department with some longer delays and thus provides a unique opportunity to assess this issue. This monocentric retrospective study shows that a cutoff of 24 days allows to define 2 groups of patients with different outcomes in terms of LC. New planning CT should be considered after a maximum period of 3 weeks (ideally 2 weeks) between the planning CT and the start of the treatment. Until adaptive online radiation therapy becomes fully integrated in daily practice, the DPT should be systematically reported and tested in the different studies.
